# Magnification‐Invariant Retinal Distance Estimation for Vitreoretinal Surgery Using a Laser Aiming Beam

**DOI:** 10.1002/rcs.70113

**Published:** 2025-10-03

**Authors:** Arpita Routray, Chaniya Jaroenkunathum, Sungwook Yang, Robert Maclachlan, Jennifer Adeghate, Marjan Fooladi, Joseph Martel, Cameron N. Riviere

**Affiliations:** ^1^ The Robotics Institute Carnegie Mellon University Pittsburgh Pennsylvania USA; ^2^ Biomedical Engineering Carnegie Mellon University Pittsburgh Pennsylvania USA; ^3^ Center for Intelligent & Interactive Robotics AIRobot Institute Korea Institute of Science and Technology (KIST) Seoul Korea; ^4^ Department of Ophthalmology University of Pittsburgh Pittsburgh Pennsylvania USA

**Keywords:** robot‐assisted surgery, tremor compensation, vitreoretinal microsurgery

## Abstract

**Background:**

Virtual fixtures in robot‐assisted retinal surgery require knowledge of the position of the retina with respect to the surgical tool to be effective. Retinal surface estimation is a difficult problem due to the lack of features in the captured microscope imagery and a complex light path. A laser aiming beam attached to the tool can be easily detected in the microscope imagery and provide valuable information about the location of the surface.

**Methods:**

We propose using the area of a laser aiming beam attached to the surgical tool to determine the distance of the tool from the retina. This area was modified in accordance with the tool width to ensure independence from microscope magnification. Retinal distance is predicted using a dual Kalman filter that combines distance inferred from this metric with information from an optical tracker that tracks the position of the tool in a global coordinate system. This updates both the state and parameters of the system in parallel and allows us to predict retinal distance even with errors in initial parameters.

**Results:**

The laser metric's independence from microscope magnification is demonstrated by plotting the metric at 3 different magnifications for a number of angles. We also predict the distance of the tool from the retina for various random angles at each magnification with median errors of less than 100 μm. Finally, we predict distance at each magnification during freehand motion and validate our results using a force sensor placed underneath the phantom.

**Conclusions:**

Using the area of laser aiming beam attached to the surgical tool, our method can predict the distance of the surgical tool to the retina with errors that are acceptable for implementing virtual fixtures during robot‐assisted retinal surgery. The predicted distance is also independent of microscope magnification and can work when initial parameters are not precisely known. Future work will involve adapting this method to in vivo environments and further reduction in prediction errors.

## 1 Introduction

Retinal surgical procedures require surgeons to manipulate very delicate tissues with little room for error. For example, during epiretinal membrane surgery, to reduce the chances of recurrence, surgeons may have to remove the 10 μm thick internal limiting membrane from the retinal surface [1, 2]. An experimental procedure to treat retinal vein occlusion is retinal vein cannulation. During this procedure, surgeons are required to inject drugs into retinal veins that are less than 100 μm thick [3, 4]. The difficulty of performing these procedures is exacerbated by natural human hand tremor, which can translate to 100 μm vibrations at the surgical tooltip [5]. Additionally, surgeons have to perform these procedures in a constrained workspace, keeping the surgical tool stationary at the point of entry in the sclera. Visualisation during these procedures may also be limited to microscope imagery.

**FIGURE 1:**
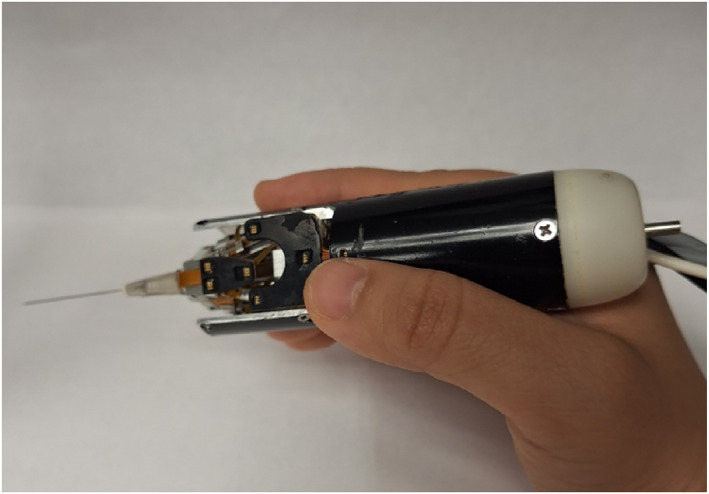
Micron, a 6‐DoF handheld manipulator for retinal surgeries.

Robotic surgery systems can aid surgeons during these demanding procedures by cancelling tremor at the surgical tooltip, providing improved visualisation, implementing motion scaling, and constraining the tool to remain within certain regions [6, 7]. Examples of robotic systems for assisting retinal surgeries include the Preceyes Surgical System [8], the JHU Steady Hand Eye Robot [9], and Micron [10]. We demonstrate our method using the handheld robotic manipulator Micron. Micron can move an attached surgical tool in a small workspace around its original position using a 6‐DoF Gough Stewart platform. The manipulator along with an exploded view of its interior is shown in Figure 1. The Micron control system cancels out hand tremor at the surgical tooltip and can also be used to enforce a stationary remote centre of motion (RCM). The position of Micron can be tracked by an optical tracking system called ‘Apparatus to Sense Accuracy of Position’ (ASAP) [11].

An essential piece of information that is required to create and enforce safeguards using such robotic systems is the distance of the surgical tool tip from the retinal surface. This knowledge can help us implement virtual fixtures that prevent the tool from penetrating beyond a certain predetermined level. This can also help us implement zones above the surface in which the motion of the surgical tool is scaled for improved control [12, 13].

**FIGURE 2:**
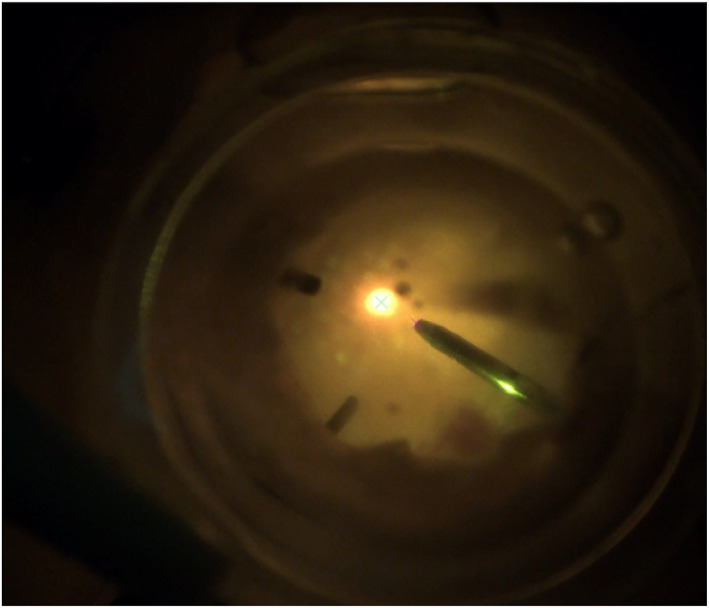
Images of the retina can be quite featureless during surgery. However, a laser spot can be clearly distinguished in these images taken during an in vivo procedure in the rabbit eye. The blue crosshair indicates the centre of the detected laser spot.

Cameras attached to the microscope above the surgical workspace are accessible sources of information about the state of the tool and the retina in real time. However, the light path from the retinal surface to the camera is complex and comprises of saline water, the cornea, the microscope lens, and a BIOM (Binocular Indirect OphthalmoMicroscope) lens. Moreover, pictures of the retina during surgery can be featureless and cloudy. Thus, many classical computer vision techniques that assume a pinhole model for the light path fail. In [14, 15], the authors estimate the distance of the surgical tool from the retina using Optical Coherence Tomography (OCT). Although OCT scans provide high resolution scans of the underlying tissue, they can be expensive to develop and have a limited range of around 2 mm. One remedy to the lack of features in retinal images is the use of a laser‐aiming beam attached to the surgical tool. Laser aiming beams are already incorporated into therapeutic lasers used on the retina, and our algorithm requires integration of such lasers with other surgical tools. As seen in Figure 2, the laser spot emanating from the tool is quite visible in images and the spot itself is very easy to detect using simple thresholding operations.

In [16], the authors predetermine the location of the retinal surface by moving the laser beam in a circle and analysing the shape of the projected ellipse. In [17], the authors study the shape of the laser spot itself to determine distance from the retina in real time. However, the distances they deal with are large compared to the range of distances that are needed in order to implement the virtual fixtures described previously. These techniques are also calibrated for a particular magnification and are bound to fail if the surgeon changes this magnification during surgery. Lastly, as the instrument tip nears the retina, the laser spot is occluded by the tool and can no longer be approximated by an ellipse. Although other laser‐based distance measurement systems have been developed [18], it is difficult to use them due to the spatial and optical restrictions inside the eye and the small distances involved.

We propose using the area of the laser as an indicator of the distance of the tool from the retina. When the tool is very close to the retina, the laser spot is mostly occluded. As the tool moves further away, the area increases due to a combination of decreasing occlusion and a larger projection area. We divide this area by the square of the tool width and the cosine of the tool angle to obtain a metric that is independent of the magnification and orientation of the tool. Finally, we use a dual Kalman filter to estimate the distance of the tool from the retina and correct for any discrepancies in model parameters, in order to avoid reliance on pre‐operative calibration.

We developed our surface estimation method using the Micron robotic system [10] and data from its optical tracker ASAP [10] is essential to the functioning of the filter. However, this method can be used by any robotic surgery system that is capable of tracking the surgical tool, which is commonly the case in robot‐assisted surgery.

## 2 Materials and Methods

We limit our investigation to cases where the tool is at angles between 60° and 75° from the horizontal. This range of angles was selected to match that observed during surgical experiments in the leporine model in vivo conducted in our laboratory. This narrow range of tool angles arises from two factors. First, the tool's motion is restricted laterally at the point of incision in the sclera, a point also commonly known as its Remote Centre‐Of‐Motion (RCM). Secondly, the tool must be visible in the microscope's field of view. Movement outside this range of angles leads to the tool moving out of the area of interest and no longer being visible in the microscope imagery. We also assume that the surface visible underneath the microscope is planar. This leads to errors of less than 10 μm when the tool tip is within 1 mm of the retinal surface.

### 2.1 Distance Metric

**FIGURE 3:**
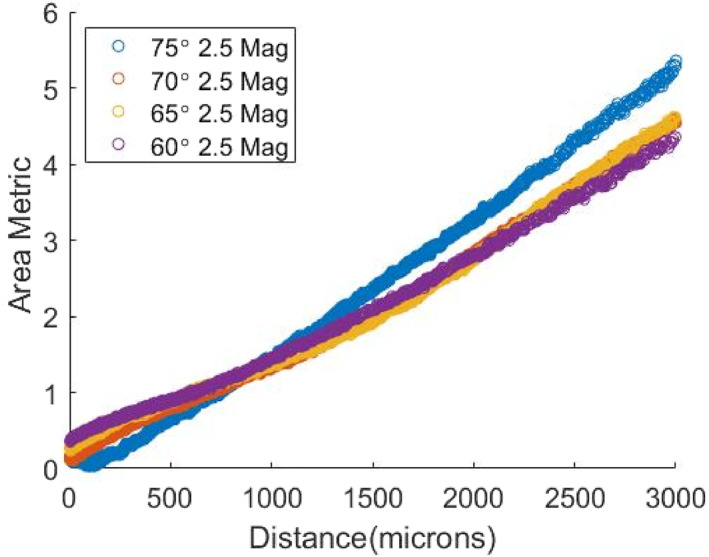
Curves for the area metric at 75°, 70°, 65° and 60° for 2.5x magnification. A linear polynomial is sufficient to describe the nature of this curves.

Before using the laser area in the measurement equation of the dual Kalman filter, we normalise it to ensure its independence from microscope magnification and tool angle. We call this normalised term the distance metric and it is defined as:

(1) $$z=\frac{a}{t^{2}\;\cos\;\theta },$$

where a is the area of the laser spot, t is the width of the surgical tool, and θ is the angle of the surgical tool to the retina. Calibration curves for this metric, measuring along the tool axis, are obtained at pre‐selected angles. Even though the normalisation factor accounting for tool angle is a simplification, over the small range of distances we are interested in, the resulting metric is approximately linear and independent of angle as seen in Figure 3. Using a more nonlinear but accurate function does not yield any significant advantages as (i) the exact geometric relationship between the tool angle and projected laser area will be affected by the complex optical conditions of the eye, and (ii) the filter may not easily converge to a correct value for highly nonlinear state and measurement equations. We thus fit a linear function to these curves to obtain an initial estimate of the system parameters.

### 2.2 Laser Spot and Tool Detection

**FIGURE 4:**
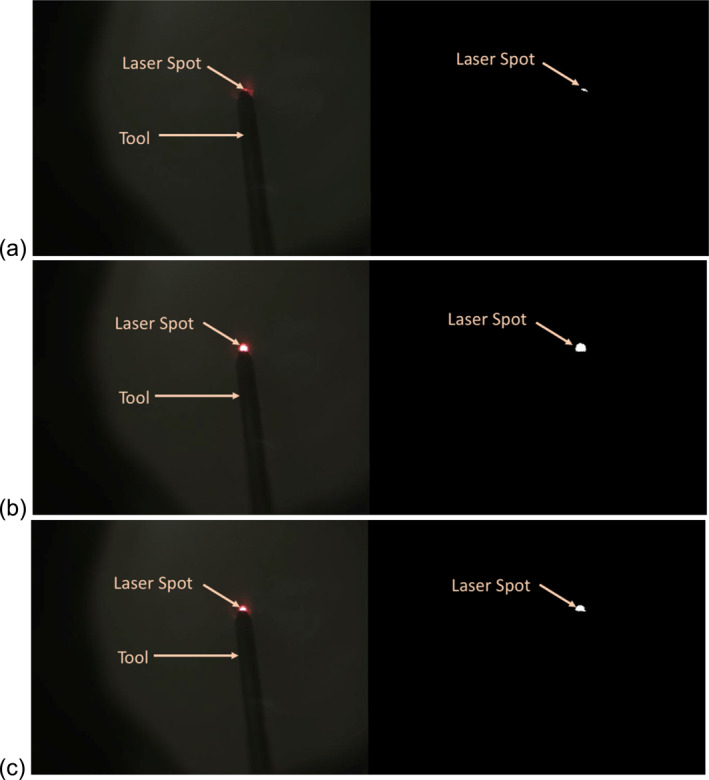
Images showing the microscope imagery (left) and the detected laser spot (right) at distances of (a) 0 μm, (b) 200 μm, and (c) 400 μm. Images were captured at a tool angle of 70° and 2.5x magnification. The laser spot's area progressively increases as the height of the tool increases.

As seen in Figure 4, the projected laser spot is much brighter than the rest of the image. Thus, a simple thresholding operation is sufficient to detect the spot. To compensate for changes in image brightness, this threshold is chosen to be a function of the tool brightness. The binary image obtained via thresholding is further processed by retaining only the largest connected area and discarding the rest. We limit our search of the tool in the image to areas adjacent to the detected laser spot. As the tool is significantly darker than the background, we detect its outline using Otsu thresholding.

### 2.3 Dual Kalman Filter

We use a dual Kalman Filter [19, 20] to simultaneously estimate the state and correct for any errors in the parameters of our system. We separate the retinal height to be estimated by the filter, h, into two terms—a constant term $h_{\text{offset}}$, and a dynamic term $h_{\text{tracker}}$. These two variables make up the state vector of the system. Micron's optical tracker ASAP can track the position and orientation of the surgical tool in a global coordinate space. Thus, we get accurate values of changes in the tool height due to tool motion from this device and these changes are tracked by the variable $h_{\text{tracker}}$. To get the correct tool height h, we add to $h_{\text{tracker}}$ an offset $h_{\text{offset}}$ that is estimated by the filter.

We define our state as

(2) $$x_{k}={\left[h_{\text{offset},k},h_{\text{tracker},k}\right]}^{T}.$$

The input to the system, u, is the measured value of $h_{\text{tracker}}$ as obtained from the tracker.

As seen in Figure 3, our metric is an approximately linear function of the tool height. Thus, our parameter vector is $P_{k}=\left[p_{0},p_{1}\right]$, where $p_{0}$, and $p_{1}$ are the coefficients of a linear polynomial that relates our distance metric $z_{k}$ to the height of the tool from the retinal surface.

Thus, our state equations are:

(3) $$x_{k}=f_{k}\left(x_{k-1},u_{k},P_{k},w_{k}\right)=\left[\begin{array}{cc} 1 & 0\\ 0 & 0 \end{array}\right]x_{k-1}+\left[\begin{array}{c} 0\\ 1 \end{array}\right]u_{k}+w_{k},$$

And,

(4) $$P_{k}=P_{k-1}+r_{k}.$$

Our measurement equation is:

(5) $$z_{k}=g_{k}\left(x_{k},u_{k},P_{k},v_{k}\right)=p_{0,k}+p_{1,k}\left(h_{\text{offset},k}+h_{\text{tracker},k}\right)+v_{k},$$

here $w_{k}$, $v_{k}$, and $r_{k}$, are independent Gaussian noise processes with zero mean and covariances $\Sigma_{w}$, $\Sigma_{v}$, and $\Sigma_{r}$. $\Sigma_{x}$ and $\Sigma_{P}$ are the covariance matrices for the state and parameter vectors respectively.

We define the following derivatives:

(6) $$A_{k}=\frac{df_{k}}{dx_{k}}=\left[\begin{array}{cc} 1 & 0\\ 0 & 0 \end{array}\right]\;B_{k}=\frac{df_{k}}{dw_{k}}=\left[\begin{array}{cc} 1 & 0\\ 0 & 1 \end{array}\right]\;C_{k}^{x}=\frac{dg_{k}}{dx_{k}}=\left[\begin{array}{c} p_{1,k}\\ p_{1,k} \end{array}\right]\;C_{k}^{P}=\frac{dg_{k}}{dP_{k}}=\left[\begin{array}{c} 1\\ h_{\text{offset},k}+h_{\text{tracker},k} \end{array}\right]\;D_{k}=\frac{dg_{k}}{dv_{k}}=1.$$

The Dual KF has the following steps:

**Table:** 

1.	${\;P}_{k}^{-}=P_{k-1}$
2.	$\Sigma_{P,k}^{-}=\Sigma_{P,k-1}^{-}+\Sigma_{r}$
3.	$x_{k}^{-}=f_{k-1}\left(x_{k-1}^{-},u_{k-1},P_{k}^{-},\bar{w}\right)$
4.	$\Sigma_{x,k}^{-}=A_{k-1}\Sigma_{x,k-1}A_{k-1}^{T}+B_{k-1}\Sigma_{\bar{w}}B_{k-1}^{T}$
5.	$L_{k}^{x}=\Sigma_{x,k-1}{\left(C_{k}^{x}\right)}^{T}{\left[C_{k-1}^{x}\Sigma_{x,k}C_{k-1}^{x,T}+D_{k}\Sigma_{\tilde{v}}D_{k}^{T}\right]}^{-1}$
6.	$x_{k}^{+}=x_{k}^{-}+L_{k}^{x}\left[z_{k}-g_{k}\left(x_{k}^{-},u_{k},P_{k},v_{k}\right)\right]$
7.	$\Sigma_{x,k}^{+}=\left(I-L_{k}^{x}C_{k}^{x}\right)\Sigma_{x,k}^{-}$
8.	$L_{k}^{P}=\Sigma_{P,k-1}{\left(C_{k}^{P}\right)}^{T}{\left[C_{k-1}^{P}\Sigma_{P,k}C_{k-1}^{P,T}+D_{k}\Sigma_{\tilde{v}}D_{k}^{T}\right]}^{-1}$
9.	$P_{k}^{+}=P_{k}^{-}+L_{k}^{P}\left[z_{k}-g_{k}\left(x_{k}^{-},u_{k},P_{k},v_{k}\right)\right]$
10.	$\Sigma_{P,k}^{+}=\left(I-L_{k}^{P}C_{k}^{P}\right)\Sigma_{P,k}^{-}$

The distance of the tip to the retina at time step k is

(7) $$h_{k}=h_{\text{offset},k}+h_{\text{tracker},k}.$$

To ensure the tool's stable motion near the retina, the parameters of the dual KF are frozen at a tool height of 1000 μm and it stops learning $h_{\text{offset}}$ at 200 μm.

### 2.4 Experimental Setup

**FIGURE 5:**
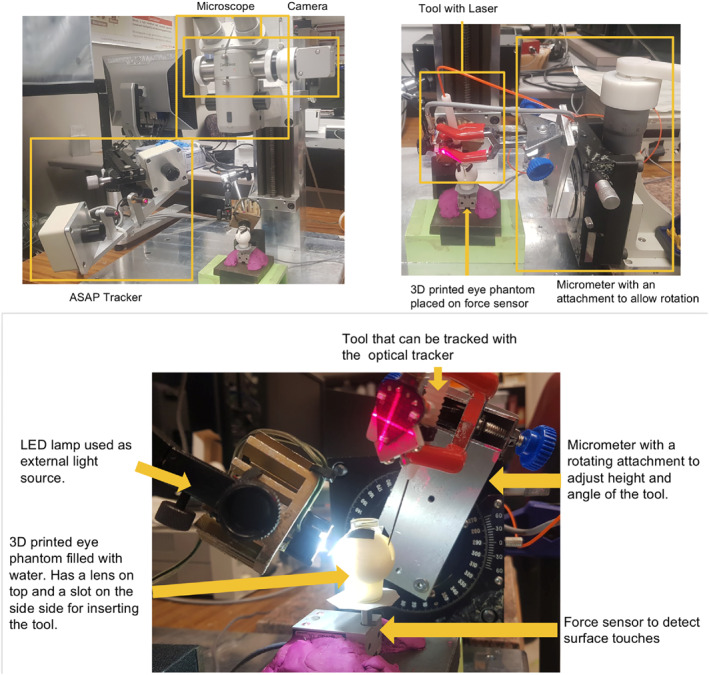
Experimental setup consisting of a linear micrometre with a rotary arrangement, surgical tool with laser, optical tracking system. microscope, and camera.

The setup for our experiments is shown in Figure 5. The work did not involve human or animal subjects and did not require ethical approval. The surgical tool is attached to a

linear micrometre that also has an arrangement for rotary motion. A spherical phantom with water was used in the experiments. A slit in the phantom allows rotation and movement of the tool using a micrometre. Although the phantom is spherical, as our method assumes that the operating region is planar, it can be used when the eye deviates from a spherical shape. Because of the slit present for controlled tool movement, the phantom was partially filled with water. However, since our method calibrates in situ, it can be used to predict retinal distance under different conditions.

Bending of the tool can lead to the tool touching the surface even after the micrometre has been raised by tens of microns. A force sensor placed below the tool helps determine the exact time at which the tool loses contact with the surface and thus can be used to estimate the tool height's zero point. Ground truth is computed from the optical tracker data as the difference between the tool's current position and its zero point.

Calibration curves to obtain an initial estimate of the parameters were recorded between 60° and 75° at intervals of 5° by slowly moving the micrometre upwards from the point of surface touch.

## 3 Results

Figure 3 shows the calibration curves for the chosen metric between 60° and 75° at intervals of 5° and 2.5x magnification. The descriptor is monotonic and fairly linear over the distances we are interested in. These curves are then used to estimate the parameters of a linear polynomial function that approximates the chosen area metric as a function of the distance of the tool from the retina.

**FIGURE 6:**
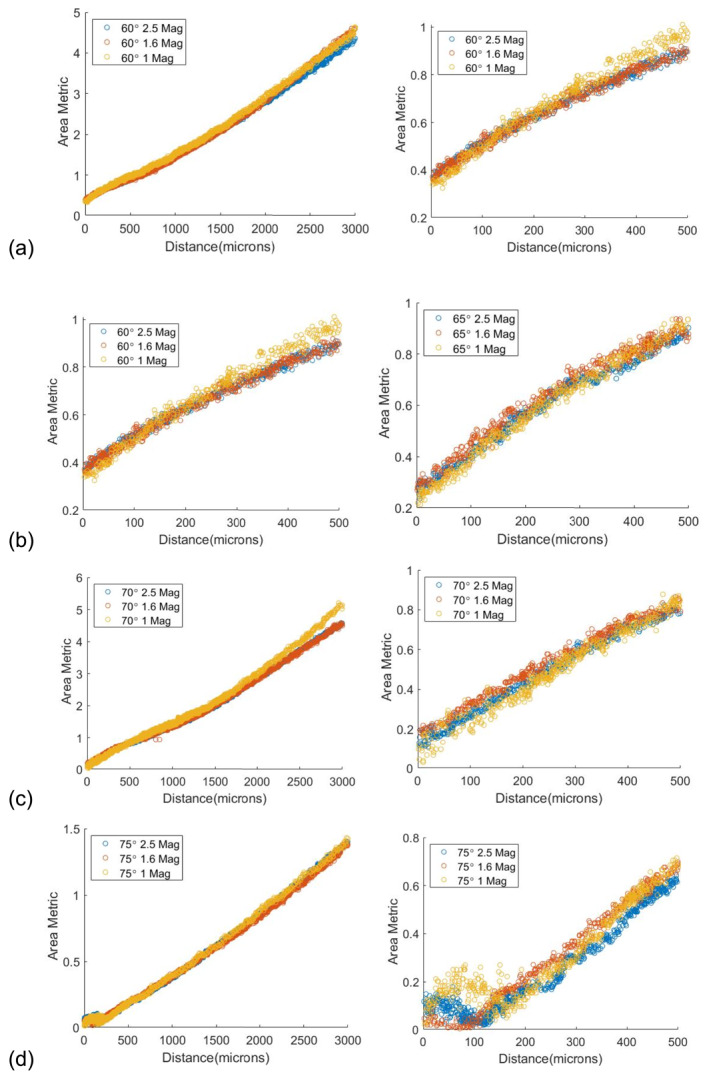
Plots of our metric at different magnifications for (a) 60°, (b) 65°, (c) 70° and (d) 75°. Our chosen metric is independent of magnification over the range of angles we are interested in.

The metric at these angles for 3 different magnifications is shown in Figure 6. The plots show that the chosen indicator is fairly independent of magnification. The indicator is also fairly independent of angle except at small distances due to effects such as occlusion from the tool.

**FIGURE 7:**
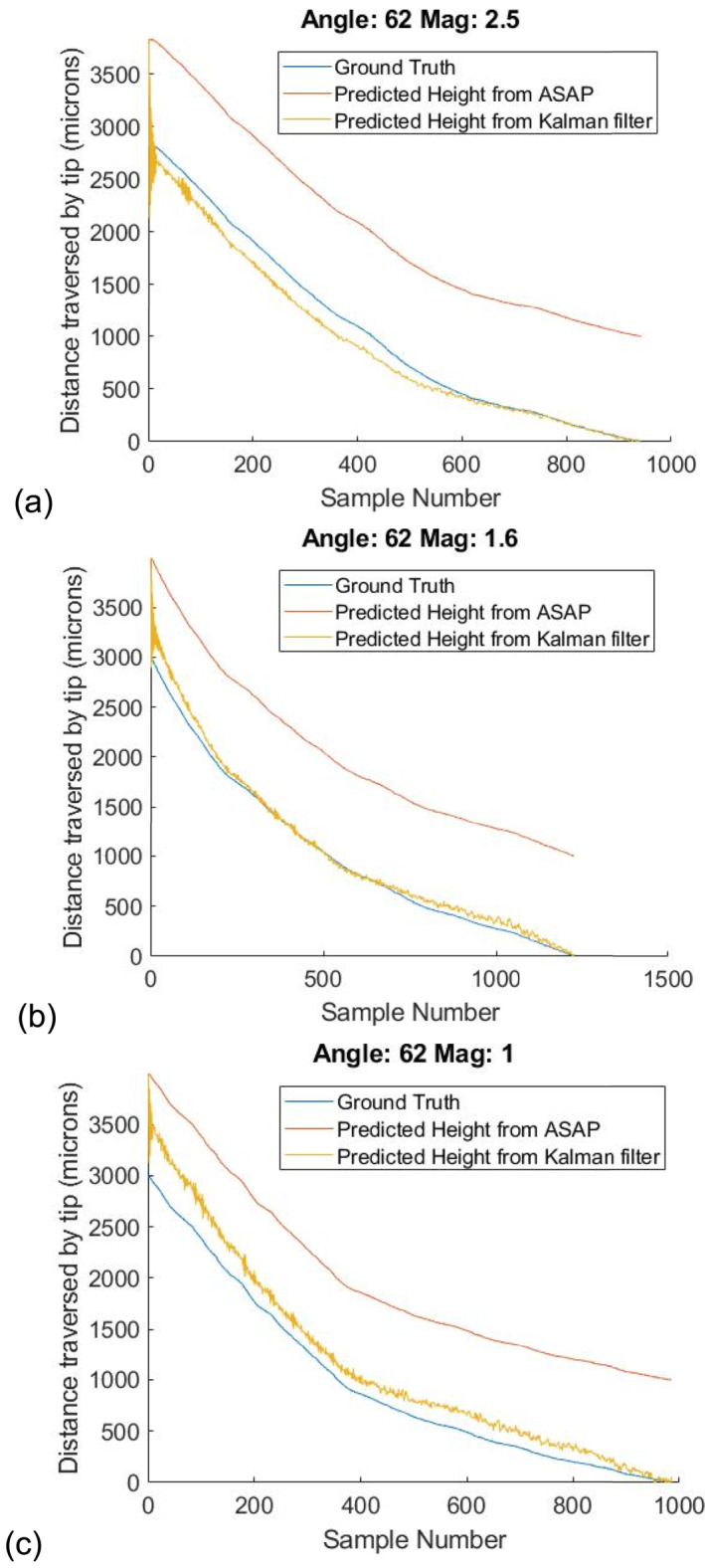
Prediction curves at 62° for (a) 2.5x, (b) 1.6x, and (c) 1.0x magnification. Median errors over distances below 300 μm are (a) 37 μm (b) 61 μm (c) 72 μm.

**FIGURE 8:**
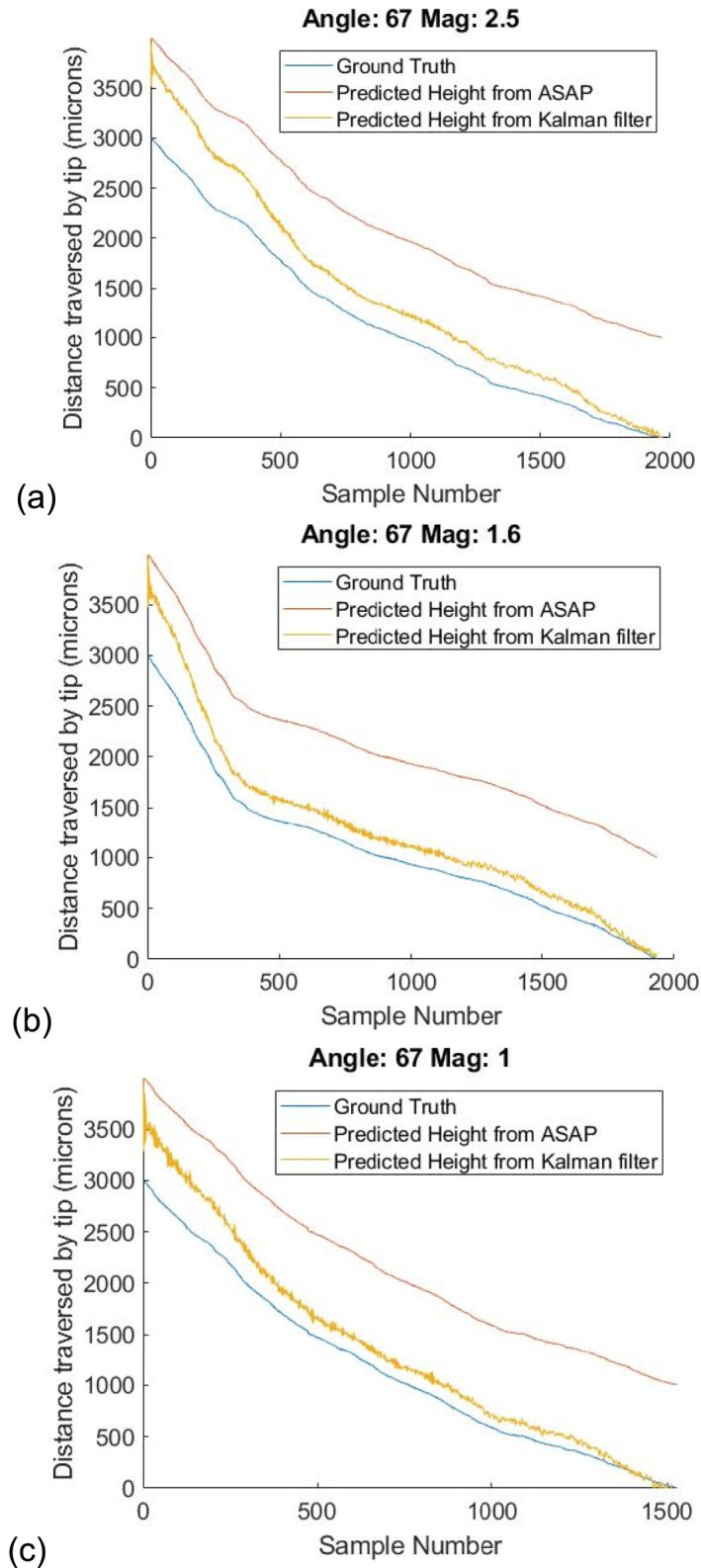
Prediction curves at 67° for (a) 2.5x, (b) 1.6x, and (c) 1.0x magnification. Median error over distances below 300 μm are (a) 51 μm (b) 32 μm (c) 43 µm.

**FIGURE 9:**
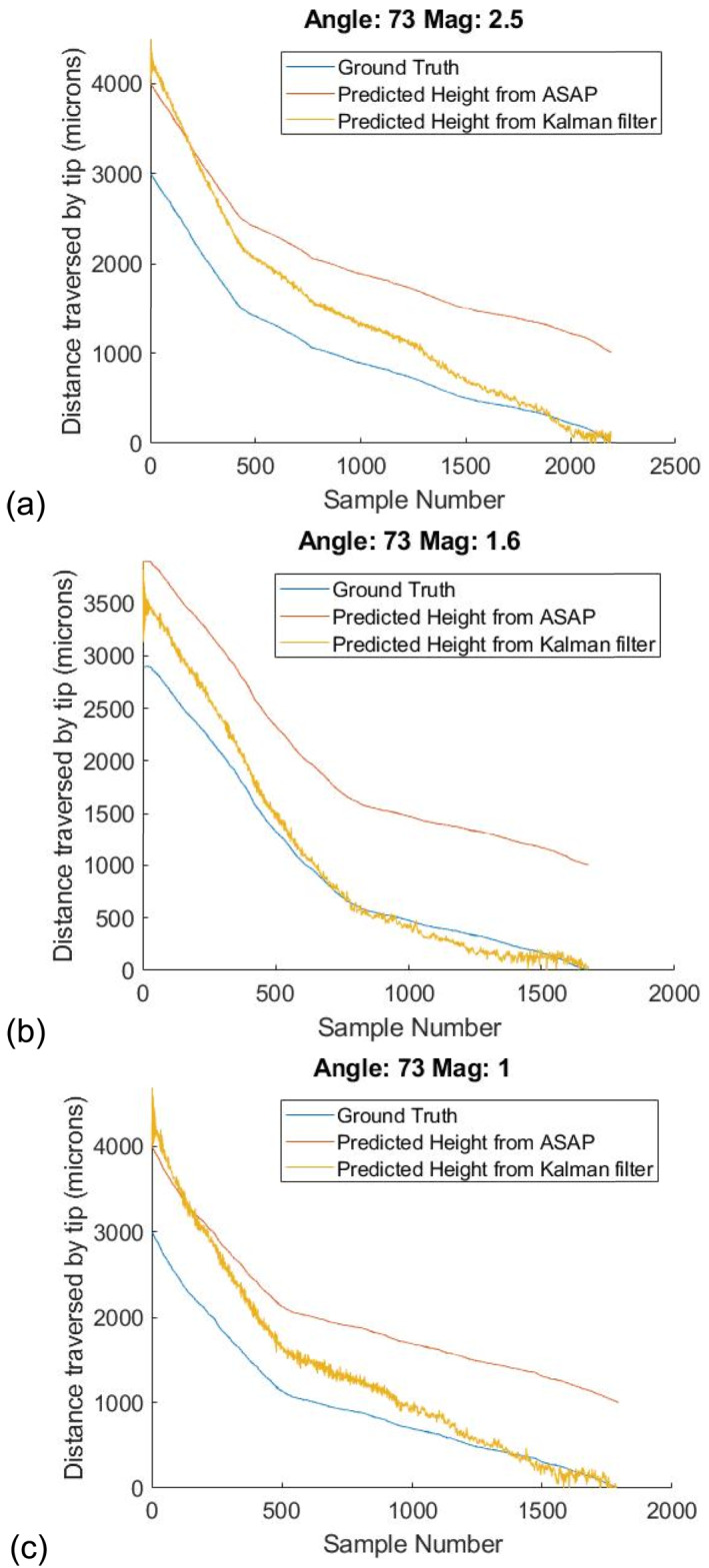
Prediction curves at 73° for (a) 2.5x, (b) 1.6x, and (c) 1.0x magnifications. Median error over distances below 300 μm are (a) 73 μm (b) 60 μm (c) 49 μm.

**FIGURE 10:**
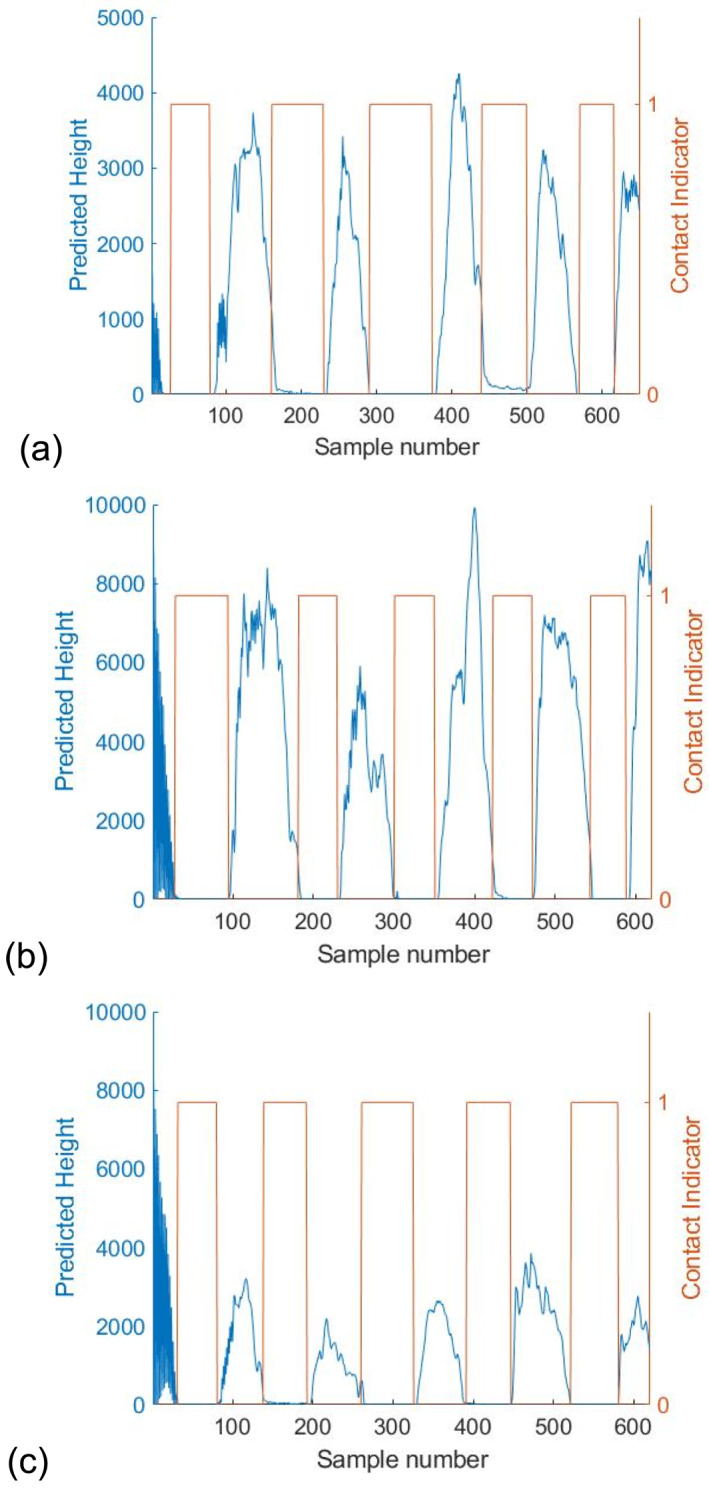
Predicted distance during freehand motion for (a) 2.5x mag, (b) 1.6x mag, and (c) 1.0x mag. The red plot highs indicate contact as inferred from the force sensor.

To demonstrate the effectiveness of the metric, we estimate distances at 3 random angles of 62°, 67° and 73° and 3 magnifications, as shown in Figures 7, 8, 9. Initial parameters for the filter are calculated from the calibration curves in Figure 5 by fitting the data at all 4 angles to a linear polynomial. For each angle, we repeat the estimation 20 times with a random deviation in the initial parameters within 30% of their true values. The median error in our estimates for the three cases is well below 100 μm in the range of distances between 0 and 300 μm. Finally, tool height is predicted during freehand motion at these 3 magnifications in Figure 10. Contact during this experiment is inferred from the readings of the force sensor placed underneath the phantom. The time of contact is determined to be when the force on the surface exceeds 70 mN.

## 4 Discussion

In this paper, we proposed to use the projected area of a laser aiming beam attached to a surgical tool to determine the distance of the tool from the retina with high accuracy. The sensitivity and monotonicity of this metric at small distances were demonstrated for various angles. We showcased the robustness of this metric to microscope magnification and used it to predict tool height using a dual Kalman filter. We also used our method to predict retinal distance at 3 angles with random deviations in the initial parameters. In every case, our filter was able to satisfactorily converge to the correct height. We also demonstrated the effectiveness of our method during freehand motion at 3 different magnifications.

Future work will involve adapting the method to work for procedures in vivo and using the distance predicted to implement virtual fixtures during these procedures. This involves finding the location of the laser spot in the microscope imagery, identifying the surgical tool, and adapting the metric calibration obtained during lab conditions to conditions in vivo. We will also aim to further reduce our errors in retinal distance estimation by including other information about the projected laser area in our filter. This includes information about the area's shape and its distance from the surgical tool. We have incorporated fibre‐optic light sources into several instruments of varied types in order to implement this technique, and will continue to do so.

Numerous factors in real surgery will make the use of this technique more difficult. Although the projected laser area is quite bright compared to its background, the light spot from the surgeon's light pipe might interfere with the laser spot. Reflections in the lens placed atop the cornea during surgery can also lead to the presence of multiple laser spots in the microscope imagery. The surgeon may also move the tool such that the laser spot flits in and out of the visible camera scope. Challenges in surgical tool detection arise from the presence of a tool shadow whose orientation and size change depending upon the illumination conditions.

Ergonomically, surgeons might be inconvenienced during the procedure due to the presence of a laser spot. To avoid this issue, we will explore using an infrared aiming‐beam instead of a laser. Additionally, in these experiments, the method stops learning the parameters of the dual Kalman filter at a tool height of 1000 μm and stops learning the state variable $h_{\text{offset}}$ at 200 μm. At closer distances, h is estimated from the optical tracker data $h_{\text{tracker}}$ and the latest value of $h_{\text{offset}}$ using Equation 7. This allows surgeons to estimate the retinal surface preoperatively and then turn off the light source attached to the tool. Thus, our method can estimate retinal distance without interfering with the surgeon's view of the workspace when contact is imminent.

There are several retinal surface estimation methods in current use that use light incorporated with the surgical tool that remain active even when the tool is very close to the retina. The authors of [21, 22] use such tools in conjunction with OCT to detect the retinal surface, and the laser used in the technique presented here need not have greater power than that used in such OCT systems. Additionally, our method only requires a few seconds to estimate the retinal surface and the light does not necessarily need to be pointed at the same spot for prolonged periods of time. This, combined with the possible use of infrared and the ability to learn the retinal surface preoperatively by only needing to reach a certain tool‐to‐retina distance, can potentially mitigate any foreseeable issues of light toxicity. Future work will involve thoroughly testing our method with regard to this issue.

At higher tool angles, the tool may severely occlude the laser spot, leading to noisy predictions. An example of this phenomenon is shown in Figure 9. Although stopping the filter learning before we reach the retinal surface helps with this issue, to completely address it, we need to be able to stop learning at even higher distances. Future work will involve incorporating more descriptors about the laser spot in the dual Kalman filter so that the system can reliably stop filter learning at higher retinal heights without sacrificing prediction accuracy.

## Author Contributions

**Arpita Routray:** conceptualization, investigation, writing – original draft, writing – review and editing. **Chaniya Jaroenkunathum:** investigation. **Sungwook Yang:** investigation. **Robert Maclachlan:** investigation. **Jennifer Adeghate:** investigation. **Marjan Fooladi:** investigation. **Joseph Martel:** investigation, writing – review and editing. **Cameron N. Riviere:** conceptualization, investigation, writing – review and editing, supervision.

## Conflicts of Interest

The authors declare no conflicts of interest.

## Data Availability

Data from this project are available at kilthub.cmu.edu.
